# Toxic Effects of Antiparasitic Pesticides Used by the Salmon Industry in the Marine Amphipod *Monocorophium insidiosum*

**DOI:** 10.1007/s00244-014-0008-8

**Published:** 2014-03-08

**Authors:** Felipe Tucca, Mauricio Díaz-Jaramillo, Gabriel Cruz, Jeannette Silva, Enrique Bay-Schmith, Gustavo Chiang, Ricardo Barra

**Affiliations:** 1 Departamento de Sistemas Acuáticos, Facultad de Ciencias Ambientales y Centro EULA-Chile, Universidad de Concepción, Concepción, Chile; 2 Laboratorio de Ecotoxicología y Contaminación Ambiental ECoA, IIMyC-CONICET, Universidad de Mar del Plata, Mar del Plata, Argentina; 3 Laboratorio de Bioensayos, Facultad de Ciencias Naturales y Oceanográficas, Universidad de Concepción, Concepción, Chile

## Abstract

The use of antiparasitic pesticides (APs) has been widely required by the salmon industry to treat diseases. The direct emission of chemicals in the seawater has produced uncertainty about the potential effects on nontarget organisms, such as crustaceans. The aim of this study was to assess the toxicity of three APs used by the salmon farm industry, such as emamectin benzoate (EB), cypermethrin (CP), and deltamethrin (DE), in the amphipod *Monocorophium insidiosum* during 10 days through whole-sediment bioassay tests. Lethal concentration by 50 % (LC_50–10d_) and biochemical responses, such as glutathione S-transferase (GST) and thiobarbituric acid reactive substances (TBARS), were measured as exposure and effects end points, respectively. Acute assays for DE (7.8 μg kg^−1^, confidence interval, CI_95%_ 5–11) and CP (57 μg kg^−1^, CI_95%_ 41–77) showed more mortality than EB (890 μg kg^−1^, CI_95%_ 672–1,171). In this study, it was possible to observe sublethal responses in amphipods after 2 days of exposure to APs. Significant induction in GST and TBARS (*p* < 0.05) were measured for CP and EB. Lower DE concentrations showed no significant biochemical responses. *M. insidiosum* was sensitive to AP concentrations at μg kg^−1^ in sediments. This information would allow considering the possible consequences of detected concentrations for APs in areas with intensive salmon farming activity.

During the last decade, the salmon industry has shown remarkable growth within aquaculture. However, in recent years, the susceptibility of salmon farms to ectoparasitic disease outbreaks has resulted in significant economic losses due to decreases in production (Johnson et al. 2004; Costello 2006; Torrissen et al. 2013). For effective mitigation, management, and control of parasites, the industry has required a wide range of antiparasitic pesticides (APs), such as chemotherapeutic treatments. Pesticides, such as emamectin benzoate (EB), avermectin and synthetic pyrethroids, cypermethrin (CP), and deltamethrin (DE), have been used to combat parasitic diseases (Burridge et al. 2010). These compounds are mainly characterized by presenting low solubility in water and high octanol–water partitioning coefficient (log *K*_ow_ between 5 and 6) such that the probability of being absorbed by suspended organic matter and being bioavailable in sediment is high (Scottish Environmental Protection Agency, SEPA 1998; Bright and Dionne 2005).

Therefore, the potential exposure and bioavailability to sediment-associated organisms, such as benthic invertebrates, may lead to deleterious effects (Ernst et al. 2001; Waddy et al. 2007; Crane et al. 2011). Likewise, the low capacity of invertebrates to detoxify or purify the quick action of compounds, such as pyrethroids on nerve cells, compared with other organisms (e.g., mammals and birds), allows to infer the selective toxicity to nontarget organisms, such as benthic crustaceans, that vulnerable to low concentrations of these pesticides (Pérez-Fernández et al. 2010).

Marine amphipods have been successfully used as ecotoxicological test organisms in sediment due to their sensitivity to a wide variety of contaminants, abundance, easy collection and laboratory manipulation, and discrete motility in addition to being an important ecological component within the benthic community (Long et al. 2001; Mayor et al. 2008; Ré et al. 2009; Prato et al. 2010). *Monocorophium insidiosum* (Crawford 1937) is a tube-forming amphipod with an extensive distribution in coasts of Europe (Mediterranean) and east coasts of the Pacific Ocean (Kevrekidis 2004; González et al. 2008). These amphipods inhabit primarily estuarine and brackish waters from infralittoral zones with a basic supply of suspended particles, microfauna, diatoms, phytoplankton, and zooplankton (Macdonald et al. 2010). Ecotoxicological tests with these amphipods have shown effective results in tests with contaminated sediments and low-sensitivity external factors, so their responses have been considered as a good toxicity indicator (Prato and Biandolino 2006).

Toxicological assessments through the use of bioassays, combined with appropriate biomarkers on marine organisms, can result in a satisfactory method for monitoring AP (Davies et al. 2001). Biomarkers allow for assessing responses at the biochemical level by providing an early warning of the potential effects of a chemical product on living organisms (Payne et al. 1987) and are thus an assessment tool for contaminated areas (Chiang et al. 2011; Díaz-Jaramillo et al. 2013b).

The antioxidant defense system plays an important role in homeostasis as well as in the detoxification of chemicals by preventing oxidative cell damage caused by reactive oxygen species (ROS), such as superoxide free radicals (${\text{O}}_{2}^{ \bullet - }$), hydrogen peroxide (H_2_O_2_), and hydroxyl radical (OH^•^). During the toxicity pathway many pesticides produce free radicals, which in turn have the ability to induce lipid peroxidation or alter the antioxidant capacity in aquatic organisms (Livingstone 2001). Oxidative stress responses, such as the activity of glutathione S-transferase enzymes (GST) and thiobarbituric acid reactive substances (TBARS), have been used as biomarkers in marine crustaceans (Schvezov and Amin 2011; Díaz-Jaramillo et al. 2013a). GST enzymes act as catalysts for oxidizing agents through the combination of xenobiotic compounds to prevent the oxidative damage and interaction of ROS with biological macromolecules, such as DNA and lipids. Lipid peroxidation of unsaturated fatty acids in phospholipids triggers further damaging effect on cell membranes, so assessments of biomarkers, such as TBARS, have been considered good indicators of membrane peroxidation (Oakes and Van Der Kraak 2003; Gorbi et al. 2008; Hellou et al. 2012).

The aim of this study was to assess the sensitivity of the marine amphipod *M. insidiosum* to AP through ecotoxicological tests in sediment by measuring acute (lethal concentration >50 % of the population [LC_50_]) and sublethal (GST and TBARS) end points at different exposure times (2 and 10 days). This study corresponds to a first ecotoxicological registration at different levels of biological organization for this amphipod.

## Materials and Methods

### Chemical Standards

Commercial standards of EB (CAS number 155569-91-8; 99.4 % purity, Pestanal), CP (CAS number 52315-07-8; 94.3 % purity, mixture of isomers, Pestanal), and DE (CAS number 52918-63-5; 99.7 % purity, Pestanal) were purchased from Sigma-Aldrich (St. Louis, USA) for toxicological testing. Analytical standards were kept at room temperature for later use.

### Sampling

Amphipods and native sediment were collected in the intertidal zone of Cocholgüe Beach, Bay of Concepcion, Chile (36°35′ S–72°58′ W). A low anthropogenic pressure characterizes this locality. Amphipods (*M. insidiosum*) were collected over 4 cm of the surface sediment with a sieve size of 500 μm, transferred to containers with seawater and fresh native sediment, stored, and transported to the laboratory. In the laboratory, the amphipods were carefully transferred to trays with fresh seawater and kept under continuous aeration until their use in toxicity tests.

The collected sediment was used as substrate in toxicity testing: It was first sieved using a mesh size of 1,000 μm, repeatedly washed to eliminate macrofauna and larger organic particles and finally dried for 24 h at 140 °C. The fine suspended particles (FSPs) washed out by the cleaning process were left to settle and suctioned with a pipette to be added back for sediment structure reconstitution at sediment-spiking time.

### Sediment Bioassay Preparation

Each standard solution corresponding to AP was diluted in acetone organic solvent due to the feasible dissolution of the active ingredient (a.i.). The solvent control contained the maximum volume of acetone in the standard solution used for assessing pesticides. Containers with 20 g of sediment were prepared and independently spiked with AP standard. Containers were mixed to achieve homogeneity and volatilization of the solvent. Treatment concentrations are reported as μg a.i. kg^−1^ of dry sediment (μg kg^−1^). Subsequently, 150 mL of oxygen-saturated fresh seawater, 3 mL of FSP, and microalgae *Dunaliella* sp. [2 mL (approximately 1.5 × 10^5^ cell mL^−1^)] were added. FSP were provided as structural substrate for amphipod tube-building and microalgae as suspended food source. Amphipods were not further fed nor received continuous aeration during the bioassay. For each test, a number of 10–12 individuals with sizes between 3 and 4 mm were incorporated.

The sedimentological characterization in the tests was performed by determining the average particle size according to the size scale proposed by Gray (1981). Coarse and fine fractions in sediment were determined by a digital decanting tube (Emery Type) and microparticle analyzer (ELZONE 282 PC), respectively. Total organic matter content was determined by the ash-free dry-weight method by incinerating the sample in an oven for 4 h at 550 °C. Test conditions related to the main physical and chemical parameters of water and sediment are listed in Table 1.

**Table 1:** Physical–chemical characteristics measured in ecotoxicological tests with marine amphipods

Parameters	Average ± Standard deviation
Dissolved oxygen (mg L^−1^)	8.23 ± 0.36
Sediment organic matter (%)	5.54 ± 0.74
Salinity (PSU)	33.0 ± 1.6
pH	7.8 ± 0.03
Temperature (°C)	12.5 ± 1.26
Photoperiod (day:night, h)	12:12
Grain size	Coarse sand

### Bioassay Procedures

#### Acute Test

To assess lethality in amphipods, five nominal concentrations were considered, which allowed for determining LC_50_ values in amphipods after 10 days of exposure (United States Environmental Protection Agency, USEPA 1994). Previously, a preliminary test was performed through a wide range of concentrations to determine the definitive test. The nominal concentrations for each AP are listed in Table 2. Dead and immobile amphipods were registered.

**Table 2:** Nominal concentrations of antiparasitic pesticides used in the acute toxicity test

Treatments	Antiparasitic pesticide (μg a.i. kg^−1^ sediment)		
	EB	CP	DE
Control	0	0	0
1	51.2	0.24	0.8
2	128	2.7	2.7
3	320	30	9
4	800	100	30
5	2,000	330	100

#### Sublethal Test

Biochemical responses were made from the results obtained in acute tests, in which the LC in 1 % of the species tested was defined. Each test consisted of three different concentrations (five replications each) under the lethality threshold through a dilution factor of 0.5 (EB = 25, 50, and 100 μg kg^−1^; CP = 2, 4, and 8 μg kg^−1^; and DE = 0.025, 0.05, and 0.1 μg kg^−1^). Exposure times considered for each test were initial time (*t*_0_), 2 days (*t*_2_), and 10 days (*t*_10_).

Amphipods (*n* = 10–12) were pooled to obtain a reasonable amount of tissue for the biochemical analyses. GST enzyme activity was determined according to Habig and Jakoby (1981) protocol, and proteins were analyzed according to Bradford (1976). Briefly, the tissue was weighed and homogenized (1:10 w/v) in cold sucrose buffer (20 mM Tris–base, 1 mM ethylene diamine tetraacetic acid, 1 mM dl-dithiothreitol, 500 mM sucrose, and 150 mM KCl) with pH adjusted to 7.6 (Geracitano et al. 2002). As a protease inhibitor, phenylmethylsulfonylfluoride (PMSF) solution was used in the ratio 5 mL of sucrose buffer to 5 μL PMSF. Homogenates were centrifuged at 10,000 rpm for 30 min (4 °C), and the supernatant was collected and stored at −80 °C for later use. GST activity (nmol min^−1 ^mg^−1^ protein) was measured through the combination of 1 mM of glutathione and 1 mM 1-chloro-2,4-dinitrobenzene at 340 nm.

For TBARS analysis, amphipod pools were homogenized in 1.15 % KCl solution, which contained 35 μM of butylated hydroxytoluene in the ratio of 0.01 g of tissue to 90 μL of homogenization solution. Homogenates were stored at −80 °C for later analysis. Measurements were performed by fluorometric analysis (*λ*_excitation_ = 515 nm and *λ*_emission_ = 553 nm) for determining TBARS using tetramethoxypropane as standard (Oakes and Van Der Kraak 2003).

### Statistical Analysis

Lethal toxicity calculations (LC_50–10d_) were analyzed using the PROBIT regression model (USEPA 1988) and trimmed Spearman–Karber (Hamilton et al. 1977). The latter analysis was used when the data showed no normal distribution and nonparametric analysis was required.

For biochemical responses, significant differences were evaluated through analysis of variance among treatments using Newman–Keuls test (*p* < 0.05). Differences between solvent control and treatments were considered. Previously, the assumption of normality and homogeneity of data were analyzed; however, those data that showed no normal distribution were analyzed using Kruskal–Wallis nonparametric test (Di Rienzo et al. 2010).

## Results

### Acute Tests

**Fig. 1:**
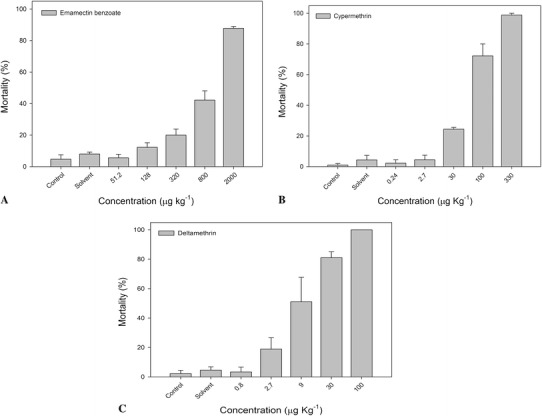
Percentage of lethality (LC_50–10d_) in *M. insidiosum* against pesticide exposure. **a** Emamectin benzoate. **b** Cypermethrin. **c** Deltamethrin

Mortality percentage in *M. insidiosum* during exposure to AP is shown in Fig. 1. Less than 10 % mortality was observed in controls with a range between 4 and 8 % lethality for the solvent control. AP testing showed that EB had the highest LC_50_ at a concentration of 890 μg kg^−1^ (95 % confidence interval (CI_95%_) = 672–1,171) contrary to what happened with CP and DE pyrethroid compounds, in which there was a greater lethality in tested amphipods with 57 μg kg^−1^ (CI_95%_ = 41–77) and 7.8 μg kg^−1^ (CI_95%_ = 5–11), respectively (Table 3). The average percentage in the variation coefficient (CV) obtained in acute tests (*n* = 3) indicates values <33 %, which is considered acceptable for ecotoxicological tests (USEPA 1991). Considering a threshold of lethality in 1 % of the test organisms, it was possible to define the concentrations for sublethal responses in amphipods (Table 3).

**Table 3:** Summary results of acute ecotoxicological tests using *M. insidiosum*

Pesticides	LC_50–10d_ (μg kg^−1^)	95 % CI		% CV (*n* = 3)	LC_1–10d_ (μg kg^−1^)
		Lower	Upper		
EB	890	672	1,171	19	230
CP	57	41	77	7	11
DE	7.8	5	11	2	0.4

LC_50–10d_ and LC_1–10d_ are lethal concentrations of 50 and 1 % after 10 days of exposure to AP, respectively

### Sublethal Tests

**Fig. 2:**
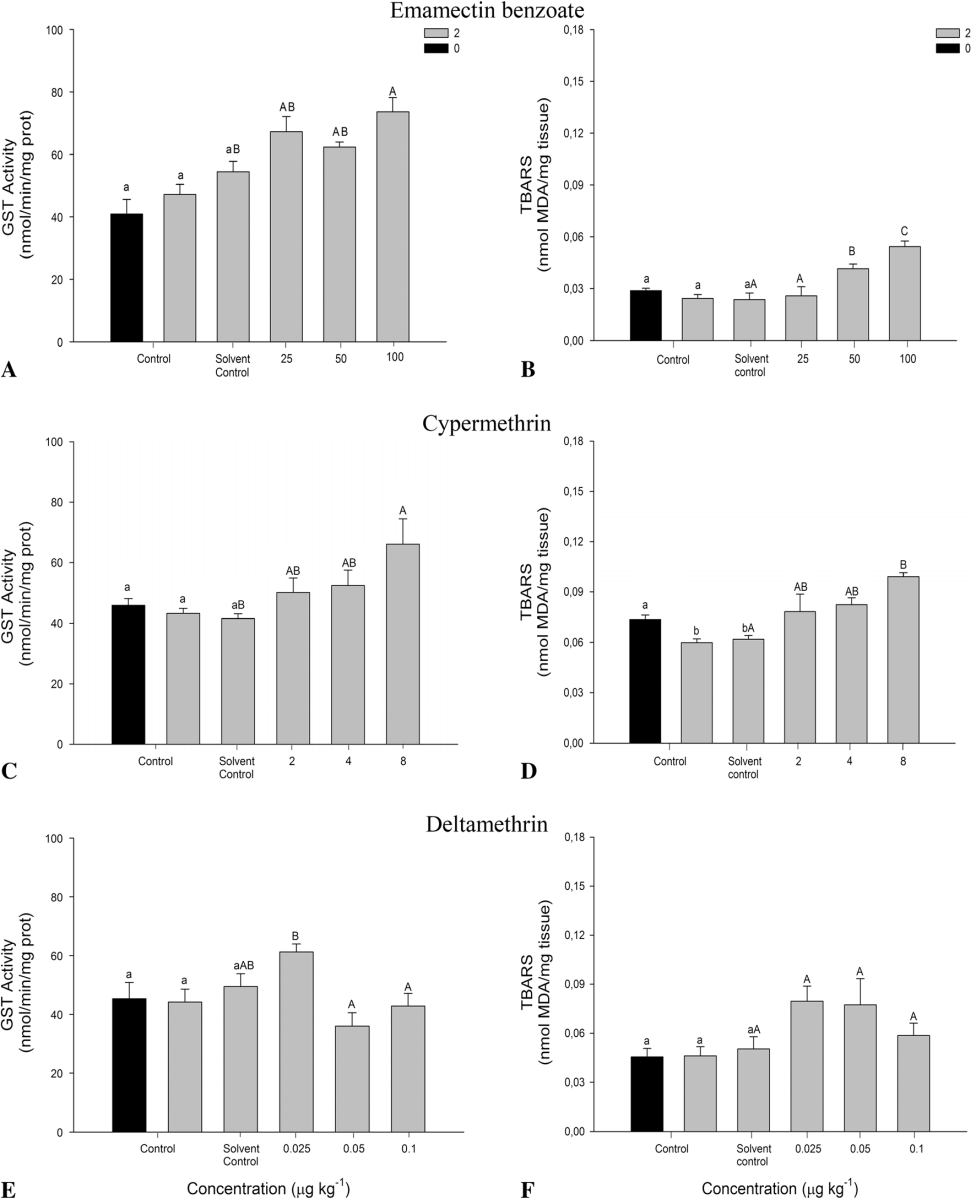
Activity values of glutathione S-transferase (**a**, **c**, **e**) and thiobarbituric acid reactive substances (**b**, **d**, **f**) in *M. insidiosum* exposed to antiparasitic pesticides for 2 days. *Lower-case letters* correspond to significant differences between controls (*black* *t*_0_, *gray* *t*_2_) and the solvent control, whereas *capital letters* indicate significant differences between the solvent control and treatments. Different *letters* show differences (*p* < 0.05)

Biochemical responses were observed in *M. insidiosum* after 2 days of exposure to pesticides (Fig. 2a–f). No significant differences were found between controls for GST activity in each of the tests with AP. However, a significant difference was observed between controls for TBARS with the CP pesticide with a greater level detected in the initial control (*p* < 0.05; Fig. 2d). For biochemical analysis between the solvent control and treatments, a significant induction can be distinguished in GST activity for 100 μg of EB kg of sediment (*p* < 0.05; Fig. 2a). Likewise, a significant increase of TBARS was observed at 50 μg kg^−1^ in the amphipods tested (*p* < 0.05; Fig. 2b). Moreover, tests with CP showed significant differences between the solvent control and GST activity at 8 μg kg^−1^ (*p* < 0.05; Fig. 2c) with a progressive increase at greater concentrations. Similarly, a TBARS increase was observed in amphipods after 2 days of exposure to CP (*p* < 0.05; Fig. 2d). In contrast, DE registered a slight increase in GST activity at the lowest exposure concentration (0.025 μg kg^−1^) compared with other treatments, but it showed no significant differences with the solvent control. Equivalently, TBARS showed no differences between treatments and control (Fig. 2e, f).

**Fig. 3:**
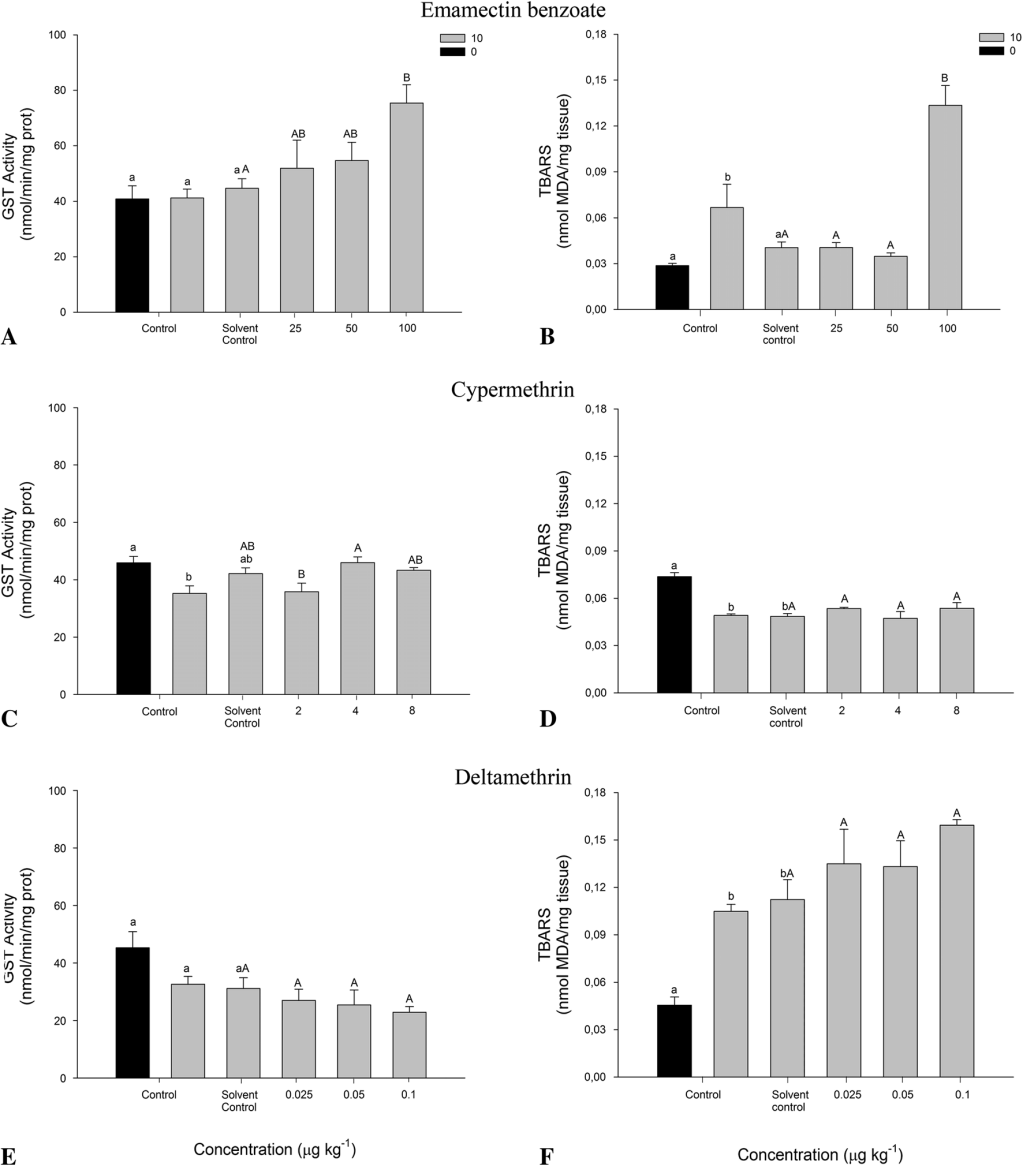
Activity values of GST (**a**, **c**, **e**) and TBARS (**b**, **d**, **f**) in *M. insidiosum* exposed to antiparasitic pesticides for 10 days. *Lower-case letters* correspond to significant differences between controls (*black* *t*_0_, *gray* *t*_10_) and the solvent control, whereas *capital letters* indicate significant differences between the solvent control and treatments. Different *letters* show differences (*p* < 0.05)

Significant differences were observed between controls and treatments for GST activity and TBARS after 10 days of exposure to AP (*p* < 0.05; Fig. 3a–f). Amphipods exposed to EB exhibited a significant induction of GST at 100 μg kg^−1^ compared with the solvent control (*p* < 0.05; Fig. 3a). Similarly, a significant increase in TBARS was reported (*p* < 0.05; Fig. 3b). In contrast, tests with CP and DE pyrethroid registered no differences for GST and TBARS activity with respect to the solvent control (Fig. 3e, f).

## Discussion

The sensitivity of *M. insidiosum* to AP varies according to the active element to which it is exposed, with pyrethroid compounds, such as CP and DE, having more effects on *M. insidiosum* than EB avermectin. Table 4 lists a summary of sediment ecotoxicological studies for different species of marine benthic invertebrates exposed to pesticides.

**Table 4:** Sediment ecotoxicological studies for EB and CP in marine invertebrates

Pesticides	Species	Types	Concentrations (μg kg^−1^)	Observations	References
EB	*C. volutator*	Amphipod	153	LC_50–10d_	Mayor et al. (2008)
			193	LC_50–10d_	SEPA (1999)
	*H. diversicolor*	Polychaete	1,368	LC_50–10d_	Mayor et al. (2008)
	*A. marina*	Polychaete	110	LC_50–10d_	SEPA (1999)
	*M. insidiosum*	Amphipod	890	LC_50–10d_	This study
CP	*C. volutator*	Amphipod	5	LC_50–10d_	Mayor et al. (2008)
			42	LC_50–10d_	Milson (1999)
	*P. pugio*	Shrimp	10	LC_50–10d_	Clark et al. (1987)
	*M. insidiosum*	Amphipod	57	LC_50–10d_	This study

Toxicity data for EB organic compound (LC_50_ = 890 μg kg^−1^) obtained in this study suggests an LC_50_ greater than that reported in the literature for other marine amphipods. Investigators, such as Mayor et al. (2008), reported an LC_50–10d_ of 153 μg kg^−1^ for the marine amphipod *Corophium volutator* through ecotoxicological tests with the commercial formulation SLICE (antiparasitic). Similarly, SEPA (1999) found an LC_50_ of 193 μg kg^−1^ for the same species. In contrast, other marine invertebrates, such as polychaete worms, have exhibited different sensitivities to EB during toxicity testing with sediment. Reports for the species *Hediste diversicolor* indicate an LC_50_ >1 mg kg^−1^; however, lethality in the order of 110 μg of EB kg sediment was determined for the polychaete *Arenicola marina*, thus showing a wide variability among species. However, SEPA (1999) has shown *A. marina* to be highly sensitive compared with other organisms tested in sediment with EB.

In contrast, an LC_50_ of 57 μg kg^−1^ was reported for CP, which is similar to the results of other studies with the amphipod *C. volutator*, in which an LC_50_ of 42 μg kg^−1^ was found (Milson 1999). Tests performed in other crustaceans, such as the shrimp *Palaemonetes pugio*, have shown greater sensitivity (Clark et al. 1987). Through acute tests performed in amphipod *C. volutator* with antiparasitic commercial product EXIS, Mayor et al. (2008) showed that CP, as an a.i. in its formulation, is 11 times more toxic than those observed in this study (Table 3). The difference obtained in results of this study for CP and EB compared with other toxicological studies with amphipods could be explained due to uneven loads of organic matter in the sediment or to poor homogenization of pesticides in this substrate, which would prevent proper distribution and bioavailability for amphipods (Maund et al. 2002; Allen et al. 2007).

Greater sensitivity was observed with the DE pyrethroid compound on the amphipods tested after 10 days, in which an LC_50_ of 7.8 μg kg^−1^ was reported. Several studies have reported acute toxicity of DE on marine invertebrates in water showing lethal levels in the order of ng L^−1^ (Ferrero et al. 2001; Adam et al. 2010; Oliveira et al. 2012). However, no information could be found on sediment toxicological tests with which to compare the results obtained in this study. Meanwhile, the high mortality of amphipods against DE can be explained by the significant toxic selectivity of this pesticide on invertebrates, mainly by the rapid and effective action exerted on the central nervous system and other tissues, thuds affecting cell transmission of organisms exposed to very low doses.

AP assessment through responses at the biochemical level can be a tool to measure the effects at a lower level of organization on nontarget organisms, which may respond to chemotherapies performed by the salmon industry (Davies et al. 2001). In our study, the role played by GST enzyme activity in the detoxifying process of the species *M. insidiosum* shows significant increases in the antioxidant defense against EB pesticide during 2 and 10 days of exposure (Figs. 2, 3). TBARS increases were observed even during GST enzyme action. This increase in lipid peroxidation on the tested organisms could be due to a failed antioxidant defense by GST enzymes when exposed to 100 μg of EB kg of sediment. In contrast, the prolonged antioxidant response in amphipods can be due to properties such as the high persistency of EB in sediment (>175 days) and high adsorption capacity to the particulate material, so that its presence after 10 days could manifest the measured behavior (SEPA 1999). Few studies of oxidative stress in invertebrates have been reported for the EB pesticide. However, significant inductions in GST activity have been reported in marine organisms, such as *Salmo salar* (Olsvik et al. 2008), the main product of cultivation in the salmon industry.

Pyrethroid compounds are a group of pesticides with a high capacity to disrupt the antioxidant capacity, producing free radicals and lipid peroxidation (Abdollahi et al. 2004). Investigators, such as Davies et al. (2001), have indicated that GST enzymes may act as a suitable indicator of exposure to CP within an enzyme-detoxification system. Increases in GST enzyme activity and effects on lipids at 8 μg of CP kg of sediment have been observed in this study after the amphipods were exposed for 2 days. However, measurements at 10 days showed no significant responses. Results may indicate that short-term exposures can provide greater reliability of the data obtained. Similarly, in a study performed by Gowland et al. (2002), it was concluded that GST inductions on the crab *Carcinus maenas* required short exposure times (24 h) to assess possible effects against exposure to CP.

According to the results obtained with DE, no significant detoxifying activity was observed by *M. insidiosum* against tested nominal concentrations; however, a slight increase in GST at 0.025 μg kg^−1^ was observed, possibly due to a disruption of the homeostatic compensatory mechanisms under the toxicological threshold before achieving the equilibrium. These behaviors have been mentioned within the field of ecotoxicology with the concept of hormesis (Calabrese and Baldwin 2001; Calabrese 2008). The reasons why there were no significant responses with the DE pyrethroid are not clear; however, the use of nominal concentrations, in addition to the small volume applied in sediment, may overestimate the concentrations assessed. Against the result obtained for DE studies, a failed antioxidant protection by GST enzymes in marine crustaceans has been observed when DE concentrations increase, thus triggering high levels of lipid peroxidation (Oliveira et al. 2012) as well as short-term oxidative damages (Dorts et al. 2009). It has been mentioned that many invertebrate responses to toxic compounds are determined by environmental factors, such as pH, temperature, and salinity, among others, which could affect acute responses at the biochemical level (Allen et al. 2007; Tu et al. 2012). However, this thought has been primarily discarded due to minimal variation of measured laboratory conditions.

Davies et al. (2001) has discussed the ineffectiveness of small crustaceans as assessment organisms at the biochemical level for chemotherapeutic compounds used by the salmon industry. However, in our study, antioxidant processes were measured for the amphipod *M. insidiosum*, and biochemical responses to sediments contaminated with metals have been reported in other amphipods as well (Schvezov and Amin 2011).

From the point of view of risk assessment and environmental relevance, AP levels found in sediment, within a radius of 100 m around net pens, have reflected concentrations in the range of 14–44 μg kg^−1^ for EB and 0.49 μg kg^−1^ for CP (SEPA 2007, 2011; F. Tucca, personal communication). Other researchers have identified concentrations of CP between 8.27 and 71.9 μg kg^−1^ in sediment of marine–estuarine areas in northeast Spain (Feo et al. 2010). No studies have found detectable DE concentrations in sediment. Consequently, EB measured concentrations are under an order of magnitude according to the levels of acute and sublethal toxicity reported for *M. insidiosum* in our study. However, the EB potential to persist and accumulate in the sediment, considering periods of consecutive treatments in salmon, can present a worst-case scenario that may result in greater levels that generate toxic side effects. In contrast, sediment values reported for CP could trigger potential environmental risks or be mostly susceptible to the action on amphipods or other marine benthic invertebrates. The lack of ecotoxicological information and environmental studies for DE in marine sediment needs greater focus of attention to generate more data that allow a proper risk assessment of this pesticide on benthic marine invertebrates (Fairchild et al. 2010).

## Conclusion

Sediment bioassays performed with the amphipod *M. insidiosum* showed responses at different levels of the biological organization and appear to be a candidate for ecotoxicological studies. Through the experimental method, it was possible to obtain contrasting results in relation to other marine organisms exposed to AP, which showed greater sensitivity to pyrethroid pesticides, such as CP and DE.

Sublethal responses, such as induction in GST activity and lipid peroxidation, were affected by EB and CP in the short-term exposures, whereas concentrations tested with DE showed no significant antioxidant activity. Thus, biochemical responses may be unclear after a longer exposure time.

In relation to concentrations measured in marine sediment, data showed that AP could cause a potential risk against the scenario of the intensive application of pesticides, in which low levels, mainly pyrethroids, would generate adverse consequences on *M. insidiosum* or other nontarget organisms in areas with aquaculture activity. Future studies in sediment require greater attention on highly toxic pesticides such as DE.
